# Airborne occupational exposures and inflammatory biomarkers in the Lifelines cohort study

**DOI:** 10.1136/oemed-2020-106493

**Published:** 2020-08-07

**Authors:** Md. Omar Faruque, Judith M. Vonk, Ute Bültmann, H. Marike Boezen

**Affiliations:** 1 University Medical Center Groningen, Department of Epidemiology, University of Groningen, Groningen, The Netherlands; 2 University Medical Center Groningen, Groningen Research Institute for Asthma and COPD (GRIAC), University of Groningen, Groningen, The Netherlands; 3 Department of Health Sciences, Community and Occupational Medicine, University of Groningen, Groningen, The Netherlands

**Keywords:** international occupational health, epidemiology, occupational health practice

## Abstract

**Introduction:**

Inflammatory biomarkers are associated with negative health outcomes. In this study, we investigated the associations between airborne occupational exposures and levels and changes in inflammatory biomarkers.

**Methods:**

We included 79 604 adults at baseline from the Lifelines cohort of which 48 403 (60.8%) subjects were followed for a median of 4.5 years. Airborne occupational exposures at the current or last-held job at baseline were estimated with the occupational asthma-specific job-exposure matrix. Both in cross-sectional and longitudinal analyses, we used linear regression models (adjusted for age, sex, education, monthly income, body mass index, smoking, pack-years, asthma and anti-inflammatory medication) to investigate the associations between airborne occupational exposures (allergens, reactive chemicals, pesticides and micro-organisms) and inflammatory biomarkers (C reactive protein (CRP), eosinophils and neutrophils).

**Results:**

In the cross-sectional analyses, exposure to allergens, reactive chemicals and micro-organisms was associated with a lower (Log) CRP level (B(95% CI)=−0.05 (−0.08 to −0.02),–0.05(−0.08 to −0.02) and −0.09(−0.16 to −0.02), respectively). Likewise, exposure to allergens, reactive chemicals, pesticides and micro-organisms was associated with a lower (log) neutrophils count (−0.01 (−0.02 to −0.01), −0.01 (−0.02 to −0.01),–0.02 (−0.04 to −0.01) and −0.02(−0.03 to −0.01), respectively). No association between airborne occupational exposures and eosinophils count was found. In the longitudinal analyses, no association between airborne occupational exposures and changes in inflammatory biomarkers was found.

**Conclusions:**

At baseline, airborne occupational exposures are inversely associated with inflammation; no effect of occupational exposures on inflammation was found at follow-up. In the future studies, details of occupational exposures, such as duration of exposures and cumulative exposures, need to be included to investigate the airborne occupational exposures and inflammatory biomarkers.

**Key messages**

**What is already known about this subject?**

- So far, associations between airborne occupational exposures and inflammatory biomarkers are investigated in a specific setting and occupation.
- Studies using a robust exposure assessment tool, such as a job-exposure matrix, are lacking in the general population.

**What are the new findings?**

- Inconsistent with previous findings, this general population-based study yielded that airborne occupational exposures were associated with a lower level/count of inflammatory biomarkers while no association was found between airborne exposures and changes of inflammatory biomarkers over time.

**How might this impact on policy or clinical practice in the foreseeable future?**

- Future studies should consider the total duration of exposure, cumulative exposure, age of first exposure and time since last exposure to detect the effects of airborne occupational exposures on inflammation over the life course.

## Introduction

Inflammatory biomarkers, such as C reactive protein (CRP), are associated with both morbidity and mortality in the general population.^1^ Inhalation of occupational exposures, for example, organic dust, triggers immune or inflammatory responses.^2^ Airborne occupational exposures account for 15%–20% of the population-attributable risk of chronic obstructive pulmonary disease.^3^ Thus, inflammatory biomarkers may provide valuable information about future health outcomes among workers, and this information may also be used to implement preventive measures.

So far, the studies that have investigated the association between airborne occupational exposures and inflammatory biomarkers focused on individual exposures in a specific occupation^4 5^ These types of studies are helpful in investigating health problems for specific settings and workers. However, due to limited sample sizes, limited clinical parameters and a limited collection of lifestyle factors, these studies often do not control for confounders of the exposure–outcome associations. In addition, the findings of these occupation-specific studies are not generalisable, and the occupational exposures are estimated using workers’ self-reported exposure. Often workers struggle to recall detailed information on working conditions many years back (recall bias), and in many instances, they link their disease condition with previous exposure (reporting bias). An objectively constructed job-exposure matrix (JEM) is a more robust tool in estimating occupational exposure and eliminating recall, reporting and differential misclassification bias.^6^

Therefore, in the current study, we investigated the association between airborne occupational exposures, assessed with a JEM, and the baseline level and longitudinal changes of inflammatory biomarkers (CRP, eosinophils and neutrophils) in the large population-based Lifelines cohort study.

## Methods

In this study, we included 79 604 adults from the Lifelines cohort study.^7^ From 2006 to 2013, baseline data were collected, and the first follow-up visit was performed after a median of 4.5 years (range:1.8–8.8 years).

Airborne occupational exposures were estimated using self-reported current or last held job title from the baseline questionnaire. The Occupational asthma-specific job-exposure matrix (OAsJEM)^8^ was linked to the baseline job title to estimate occupational exposure into no, low or high exposure categories (0/1/2). Four occupational exposure groups were created: allergens (animals, flour, house dust mites, storage mites, plant mites, enzymes, latex and fish); reactive chemicals (high-level chemical disinfectants, isocyanates, acrylates, epoxy resins, persulphates, aliphatic amines and bleach); pesticides (herbicides, insecticides and fungicides) and micro-organisms (moulds and endotoxin). These occupational exposures were dichotomised into no exposure and any exposure. The ‘no exposure’ group consisted of subjects who were not exposed to any of the 30 occupational agents in the OAsJEM. In the ‘any exposure’ group, low and high exposure were combined. The self-reported job titles were coded according to the International Standard Classification of Occupations-08,^9^ and the coding was performed by a Computer-Assisted Structured Coding Tool (CASCOT).^10^ We selected subjects with a CASCOT score ≥60. We additionally reviewed all job titles and recoded these manually if necessary to achieve accurate job coding.

Details on laboratory procedures to measure the inflammatory biomarkers are given elsewhere.^11 12^ The inflammatory biomarkers (CRP, eosinophils and neutrophils) and the residuals of the regression analyses were not normally distributed at baseline, so we used the natural log (ln)-transformation to obtain a normal distribution. In the cross-sectional analyses, linear regression models were used to investigate the associations between baseline occupational exposures (no exposure as reference) and log-transformed inflammatory biomarkers adjusted for age, sex (female as reference), education (low education as reference), monthly income (low income as reference), body mass index, smoking (no smokers as reference), pack-years, asthma (no asthma as reference) and anti-inflammatory medication (no medication as reference). In the longitudinal analyses, linear regression models were used to investigate the associations between occupational exposures and changes of inflammatory biomarkers and these models were additionally adjusted for the time between the baseline and follow-up visits. Changes in biomarkers were calculated as the difference in absolute level/numbers of the biomarkers between follow-up and baseline. At follow-up, CRP was measured in a very limited number of subjects (n=206) and was excluded from the longitudinal analyses.

All exposures were tested separately and were not adjusted for the other exposures due to multicollinearity. A p<0.05 was considered statistically significant (tested two sided).

To investigate if current occupational exposures have a different effect on inflammatory biomarkers than previous exposure, the analyses were stratified by active workers at baseline (currently have a paid job for at least 1 hour per week) and non-active workers at baseline (eg, retired, unemployed/looking for a job or unfit for work).

## Results

At baseline, the mean age was 44 (SD 13) years and 60.2% were female. Descriptive statistics of the study population is given in table 1.

**Table 1:** Baseline characteristics of the study population

Population characteristics	Total population at baseline, n=79 604
Age (years), mean (SD)	44 (13)
Female sex, %	60.2
Follow-up duration (years), mean (SD)	4 (1)
Body mass index (kg/m^2^), mean (SD)	26 (4)
Smoking never smokers, N(%)	35 218 (47.3)
Ex-smokers, N(%)	24 429 (32.8)
Current smokers, N(%)	14 783 (19.5)
Education low, N(%)	11 955 (15.8)
Medium, N(%)	38 958 (51.5)
High, N(%)	23 192 (30.7)
Unclassifiable, N(%)	1380 (1.8)
Monthly income low, N(%)	11 191 (15.2)
Medium, N(%)	19 922 (27.0)
High, N(%)	32 289 (43.8)
Unknown, N(%)	10 376 (14.1)
Anti-inflammatory medication, %	27.5
Asthma, %	8.2
C reactive protein (mg/dL), median (IQR)	1.20 (2.20)
Eosinophils (count ×10^6^/L), median (IQR)	150 (130)
Neutrophils (count ×10^6^/L), median (IQR)	3100 (1,390)
Δ eosinophil (count ×10^6^/L), median (IQR)	10 (80)
Δ neutrophil (count ×10^6^/L), median (IQR)	60 (1,010)
Allergens exposed, %	31.5
Reactive chemicals exposed, %	32.1
Pesticides exposed, %	8.5
Micro-organisms exposed, %	6.8

Δ-Difference in numbers of eosinophil and neutrophil between baseline and follow-up.

Education: low education (No training, primary education, lower or pre-vocational education); medium education (General secondary education, secondary vocational or professional guiding, preuniversity education); high education (Higher professional or university degree); Unclassifiable (Subjects with other than above-mentioned education).

Monthly income: low income (monthly net income ≤ €1500); medium income (monthly net income between €1500 and €2500); high income (monthly net income ≥ €2500); unknown (I don’t know/ I don’t want to say).

Smoking: never smokers (Never smoked or smoked for < 1 year); ex-smokers (Smoked for ≥ 1 yearand stopped smoking for ≥ 1 month); current smokers (Current smoker or stopped smoking < 1 month)

Anti-inflammatory medication: prescribed use of steroids and/or non-steroidal anti-inflammatory drugs.

IQR, Interquartile range; SD, Standard deviation.

In the cross-sectional analyses, exposure to allergens, reactive chemicals and micro-organisms were significantly associated with a lower CRP level (table 2). For example, subjects with allergens exposure had a 5% (exp(−0.05)=0.95; exp(CI)=0.93 to 0.98) lower CRP level compared with subjects without allergens exposure. Pesticides exposure was also associated with a lower CRP level; this finding was not statistically significant. All occupational exposures were significantly associated with a lower neutrophil count. No association between occupational exposures and eosinophil count was found.

**Table 2:** Association between occupational exposures and inflammatory biomarkers

Airborne occupational exposures	Cross-sectional analyses			Longitudinal analyses	
	(Ln) CRP	(Ln) Eosinophils	(Ln) Neutrophils	Eosinophils (10^5^/L)	Neutrophils (10^6^/L)
	B* (95% CI)	B* (95% CI)	B* (95% CI)	B* (95% CI)	B* (95% CI)
No exposure	Reference	Reference	Reference	Reference	Reference
Allergens	−**0.05 (−0.08 to −0.02**)	−0.01 (−0.02 to 0.01)	−**0.01 (−0.02 to −0.01**)	−12 (−40 to 16)	18 (−8 to 44)
Reactive chemicals	−**0.05 (−0.08 to −0.02**)	0 (−0.01 to 0.01)	−**0.01 (−0.02 to −0.01**)	−5 (−34 to 23)	−1 (−27 to 25)
Pesticides	−0.05 (−0.12 to 0.01)	−0.01 (−0.03 to 0.02)	−**0.02 (−0.04 to −0.01**)	−24 (−81 to 35)	51 (−2 to 104)
Micro-organisms	−**0.09 (−0.16 to −0.02**)	−0.03 (−0.05 to 00)	−**0.02 (−0.03 to −0.01**)	−35 (−96 to 26)	30 (−27 to 86)

Linear regression models were adjusted for baseline age, sex, BMI, pack-years, smoking, education, monthly income, asthma and medication. The longitudinal analyses were additionally adjusted for time between baseline and follow-up.

Bold: P<0.05.

*B-coefficients of the linear regression analyses.

BMI, body mass index; CRP, C reactive protein.

In the longitudinal analyses, no associations between occupational exposures and changes in inflammatory biomarkers were found.

When stratified for active and non-active workers, in the cross-sectional analyses, occupational exposures were associated with a lower CRP level and neutrophil count in active workers. No such associations were found in non-active workers. Occupational exposures were not associated with eosinophil count in both active and non-active workers. In the longitudinal analyses, occupational exposures were not associated with changes of inflammatory biomarkers in both active and non-active workers.

## Discussion

In this study, we investigated the associations between airborne occupational exposures at baseline and (1) inflammatory biomarkers at baseline and (2) changes of inflammatory biomarkers between baseline and follow-up. We found that occupational exposures were associated with a lower CRP level and neutrophil count at baseline. Occupational exposures were not associated with changes in inflammatory biomarkers between baseline and follow-up.

One explanation of our unexpected findings could be due to ‘immunological tolerance’. This phenomenon is described as a state of indifference or non-reactivity towards a substance that would normally be expected to excite an immunological response.^13^ A previous animal study found that tolerised mice (by repeated Ag inhalation) had a lower cellular infiltration (ie, eosinophils and neutrophils) compared with control mice.^14^ In line, a previous study showed that on swine dust exposure, farmers with previous biological dust exposure had lower white cells count compared with non-farmers.^15^ Therefore, we hypothesise that workers adapt better to the condition with repeated exposure to allergens/irritants over a long period. Another explanation of our negative findings could be physical activity. Previous studies showed that physical activity helps in reducing inflammation.^16^ We assume that workers with occupational exposure (eg, farmers and waste collectors) are more physically active compared with sedentary office workers, and a higher level of physical activity might reduce inflammation in exposed workers.

An alternative explanation for our unexpected findings could be the ‘healthy worker effect’.^17^ This implies that workers with immunological sensitivity (in another word allergic) to allergens, microorganisms, pesticides or reactive chemicals did not take up a job with these types of exposure, or switched to a job with less occupational exposure. As a result, only the workers who did not experience negative health effects from these exposures stayed in their exposed job.

In this study, we included a substantial number of subjects (who were extensively characterised) both at baseline (79 604) and follow-up (48 403) from the Lifelines cohort study. A general population-based JEM (OAsJEM) was used to estimate airborne occupational exposures based on self-reported current or last held job at baseline. This JEM was created objectively, and therefore, it eliminates recall and reporting bias as well as differential misclassification bias.^6^ However, non-differential misclassification bias cannot be ruled out which may attenuate the study findings.^6^ We did not assess exposure at the individual chemical or biological agent level that could be considered as a demerit of using OAsJEM. Furthermore, as it was not possible to incorporate the full job history and to estimate cumulative occupational exposure throughout the subjects’ entire careers, which could also be considered as a limitation and could have produced non-informative results. Finally, we adjusted for well-known covariates (also covariates that are available in the Lifelines cohort study) to overcome confounding effects. We did not adjust for covariates such as stress or physical workload. So, we cannot rule out the residual confounding effect of these unmeasured covariates in our analysis.

In this general population-based study, airborne occupational exposures are negatively associated with inflammatory biomarkers at baseline, but not related to changes of inflammatory biomarkers at follow-up. Future studies should consider the total duration of exposure, cumulative exposure, age of first exposure and time since last exposure to detect the effects over the life course of airborne occupational exposures on inflammatory biomarkers at baseline and changes of inflammatory biomarkers at follow-up. The results of these future studies may point towards future preventive and therapeutic measures.
